# Predictors for INR-control in a well-managed warfarin treatment setting

**DOI:** 10.1007/s11239-018-1765-4

**Published:** 2018-11-08

**Authors:** Fredrik Björck, Hayder Kadhim, Anders Själander

**Affiliations:** Department of Public Health and Clinical Medicine, Umeå University, Umeå, Sweden

**Keywords:** Oral anticoagulation, Time in therapeutic range, Atrial fibrillation, Stroke

## Abstract

Warfarin is well studied in patients with non-valvular atrial fibrillation (AF). It has low complication rates for patients achieving individual Time in Therapeutic Range (iTTR) > 70%. The risk scores SAMe-TT_2_R_2_ and PROSPER are designed to predict future TTR, but are derived from a heterogeneous population with generally low iTTR. The aim of this study was to evaluate predictors for high and low iTTR in an AF population in Sweden, where there is a generally good anticoagulation control. A retrospective register study based on Swedish warfarin dosing system AuriculA, including 28,011 AF patients starting treatment during 1 January 2006 to 31 December 2011. Complications and risk factors were analysed and related to iTTR. Mean age was 73.7 (SD ± 9.5) years, with 42.0% women. Mean CHA_2_DS_2_-VASc score (SD) was 3.6 (± 1.7). For patients with iTTR < 60% there were over three times higher prevalence of excessive alcohol consumption than for patients with iTTR > 70% (3.7% vs. 1.1%). Previous stroke were more prevalent for patients with high than low iTTR (17.1% vs. 20.3%). Concomitant comorbidities were associated with increased risk of poor iTTR. In Swedish AF patients, excessive alcohol use is clearly associated with iTTR below 60%. Patients with previous stroke are more likely to get iTTR above 70%, unlike those with concomitant disorders who more often have poor anticoagulation control. The SAMe-TT_2_R_2_-score cannot be applied in Sweden.

**Electronic supplementary material:**

The online version of this article (10.1007/s11239-018-1765-4) contains supplementary material, which is available to authorized users.

## Highlights

In a well-managed warfarin treatment setting of stroke prevention in atrial fibrillation;

- Excessive alcohol use is the strongest risk factor of iTTR < 60 %.
- Patients with previous stroke are more likely to get iTTR > 70 %.
- Patients with other comorbidities are more likely to get iTTR < 60 %.
- The SAMe-TT_2_R_2_ score does not apply.
- Instead of warfarin, DOACs could perhaps be the anticoagulant of choice, when treating patients with strong risk factors such as excessive alcohol use.

## Introduction

In Sweden, both direct oral anticoagulants (DOACs) and vitamin K antagonists (e.g. warfarin) are used as stroke preventives in atrial fibrillation (AF), and national guidelines equals those treatment options [1]. Swedish warfarin treatment quality is very high in terms of high time in therapeutic range (TTR) and low complication rates [2, 3]. A previous study on Swedish patients with non-valvular AF started on warfarin as stroke prevention, showed generally low risk of complications, especially in patients achieving TTR over 70%, concluding warfarin to be still a valid alternative for stroke prevention [4]. However, for warfarin patients with less optimal TTR levels, DOACs could provide a more safe and effective treatment option. The risk-scores SAMe-TT_2_R_2_ and PROSPER can be used to predict the future TTR level of warfarin patients [5, 6]. These scores are however deduced in a more heterogenous population with generally lower TTR-levels, and might not be valid in a high TTR setting.

The aim of this article is to clarify predictors of poor [individual TTR (iTTR) below 60%] and good (iTTR above 70%) INR-control in patients starting warfarin due to non-valvular AF in a well-managed warfarin treatment setting, with the intention to guide in future decisions of anticoagulation.

## Methods and material

Based on AuriculA, we performed a retrospective multicentre cohort study. AuriculA is a Swedish national quality register for AF and oral anticoagulation started in 2006, which now includes over 126,000 patients from 224 participating centers nationwide, both specialized anticoagulation clinics as well as primary health care centers. Approximately 50% of all patients on warfarin in Sweden are included in AuriculA. Participation in AuriculA is mostly within whole regions with no apparent selection bias [7]. Everything related to warfarin treatment for patients documented in the anticoagulation centers in everyday clinical practice is transferred to the quality register automatically once every 24 h. AuriculA also provides a clinical decision tool, aiding in the dosage of warfarin using a dosing algorithm [8]. If certain criteria are met, the algorithm presents a dose suggestion that can be accepted or manually changed.

### Cause of death register

The Cause of Death Register (CDR) includes deceased persons with a Swedish personal identity number and contains information about their age and sex, date and cause of death and whether or not autopsy was performed [9].

### Swedish National Patient Register

The Swedish National Patient Register (NPR) contains information about hospital admissions as well as visits in outpatient clinics in Sweden for all patients with a Swedish personal identity number [10]. The register was launched in 1964, with complete coverage since 1987. Currently more than 99% of all somatic and psychiatric hospital discharges are registered in the NPR. The register includes information about patient’s age and sex, dates for admission and discharge, and then registered primary and secondary diagnoses as well as codes for surgical procedures, according to the diagnose coding system International Classification of Disease, 10th edition (ICD-10).

All patients in AuriculA started on warfarin treatment due to AF during 1 January 2006 to 31 December 2011 were initially included. Patients already on warfarin treatment at study start 1 January 2006 were not included. Patients under the age of 18 years were excluded to avoid bias (one individual). Of the remaining 40,909 patients, 460 had in addition to AF valve malfunction [mitral stenosis (n = 82) or mechanical prosthetic valves (n = 378)] and were therefore excluded. Furthermore, those 11,303 patients who had no previous diagnosis recorded in the NPR were also excluded, due to lack of background data needed in further analysis. These patients had no prior hospital visit before their warfarin treatment was initiated in a primary health care setting. A final cohort of 29,146 patients, all starting warfarin treatment due to non-valvular AF with available background data, was included and followed until treatment cessation, death or end of study period at 31st December 2011. The final analysis was performed on a cohort of 28,011 patients, after an additional 1135 individuals were excluded due to lack of iTTR (caused by no or only one INR value available).

Data from AuriculA was linked to the NPR and the CDR. Complications (mortality, bleedings and thromboembolic events) and risk factors were analysed in relation to anticoagulation treatment quality as measured by iTTR. Baseline characteristics and treatment complications were retrieved from the NPR, using ICD-10 codes (Online Appendix).

Major bleeding was defined as intracranial, gastrointestinal or other bleeds requiring in-hospital care. Thromboembolic events were defined as diagnosed stroke/TIA/peripheral emboli (arterial), venous thromboembolism or myocardial infarction (see Appendix for included ICD-10 codes). Only the first complication of every subtype was included for each treatment period to reduce the risk of over-registering. For the same reason, only the main diagnosis from the NPR was used for cerebral haemorrhage or infarction as well as venous thromboembolism, while both primary and secondary diagnoses were used to define other bleeding complications, myocardial infarctions or baseline characteristics (Online Appendix). For cerebral infarction, follow up contacts soon after the index event is common and might cause double-reporting leading to diagnosis being addressed as complication rather than indication for treatment. To avoid this, a blanking period of 2 weeks was applied for ICD-codes identical to the index event in these patients.

Date of death was retrieved from the CDR. Death within 1 day from treatment cessation was included in all-cause mortality. All other complications events were counted when occurring within the treatment period. The treatment period was defined as time from start-day until stop-day of warfarin treatment or at 31st December 2011, with information derived from AuriculA.

TTR calculated according to Rosendaal et al. [11] was retrieved from AuriculA, where INR and exact treatment time is registered. If greater gap than 90 days between two neighbouring INR values, this period was excluded from that patient’s TTR-calculation. The cohort was divided in groups regarding achieved iTTR, where iTTR over 70% was considered “good anticoagulation control” according to European guidelines [12] and iTTR below 60% was considered “poor anticoagulation control”. Patients with intermediate iTTR above 60 but below 70 were not analysed further, since the aim of the study was predictors for either good or poor iTTR outcome.

### Statistical methods

Baseline characteristics were presented descriptively. Annualized incidence of complications were calculated as events per treatment year, with results expressed as percent.

Multivariable logistic regression analysis was used for calculating predictors for iTTR below 60% and over 70%.

Data was analysed using SPSS Statistics (Version 21; SPSS Inc., IBM Corporation, NY, USA), and R version 3.0.0, R Foundation for Statistical Computing, Vienna, Austria. URL http://www.R-project.org/. Confidence intervals (CI) quoted are 95%.

## Results

The final analyzed cohort of 28,011 AF patients newly instituted on warfarin had a mean age at study start of 73.7 (SD ± 9.5) years, 42.0% were women. Mean iTTR was 68.3% (± 22.4). Baseline characteristics are presented in Table 1. Mean CHA_2_DS_2_-VASc score was 3.6 (± 1.7), 19,3% had suffered a previous stroke. “Good anticoagulation control”, iTTR > 70% was found in 15,707 (56%) patients, while 7835 (28%) had a “poor anticoagulation control” defined as iTTR < 60%.

**Table 1:** Baseline characteristics of patients with atrial fibrillation started on warfarin treatment, subdivided in later achieved individual time in therapeutic range (iTTR)

Baseline variables	Total	iTTR < 60%	iTTR ≥ 70%
	n = 28,011	n = 7835	n = 15,707
Age, mean year (SD)	73.7 (± 9.5)	72.9 (± 10.4)	74.2 (± 8.9)
Male	16,261 (58.0)	4674 (59.7)	9003 (57.3)
Female	11,750 (42.0)	3161 (40.3)	6704 (42.7)
Stroke	5396 (19.3)	1341 (17.1)	3188 (20.3)
TIA	2205 (7.9)	540 (6.9)	1326 (8.4)
Hypertension	16,739 (59.8)	4973 (63.5)	9120 (58.1)
Chronic heart failure	7989 (28.5)	2500 (31.9)	4085 (26.0)
Diabetes mellitus	4978 (17.8)	1668 (21.3)	2481 (15.8)
Myocardial infarction	6030 (21.5)	1795 (22.9)	3256 (20.7)
Cancer	3292 (11.8)	1006 (12.8)	1754 (11.2)
Chronic obstructive pulmonary disease	2485 (8.9)	921 (11.8)	1120 (7.1)
Renal failure	1087 (3.9)	441 (5.6)	447 (2.9)
Excessive alcohol use	539 (1.9)	292 (3.7)	165 (1.1)
Dementia	133 (0.5)	48 (0.6)	65 (0.4)
Liver disease	239 (0.9)	100 (1.3)	105 (0.7)
History of fall	2227 (8.0)	664 (8.5)	1195 (7.6)
Anemia	1922 (6.9)	691 (8.8)	896 (5.7)
Previous major bleeding	1777 (6.3)	557 (7.1)	916 (5.8)
Previous gastrointestinal bleeding	931 (3.3)	286 (3.7)	484 (3.1)
Previous intracranial bleeding	403 (1.4)	130 (1.7)	208 (1.3)
CHA_2_DS_2_-VASc score, mean (SD)	3.6 (± 1.7)	3.6 (± 1.8)	3.6 (± 1.6)

Presented as n (%), if other not indicated

*SD* standard devation

Patients in the iTTR < 60% group had more prevalent excessive alcohol use (3.7% vs. 1.1%) and less frequently previous stroke (17.1% vs. 20.3%) compared to patients with iTTR > 70%.

All-cause mortality (7.3% vs. 1.6%), any major bleeding (5.5% vs. 1.8%) and any thromboembolism (6.8% vs. 2.8%) were more prevalent in the iTTR < 60% group compared with the iTTR > 70% group (Table 2).

**Table 2:** Complications for patients with atrial fibrillation instituted on warfarin

	All n = 28,011		iTTR < 60% n = 7835		iTTR ≥ 70% n = 15,707	
	n	% (CI 95%)	n	% (CI 95%)	n	% (CI 95%)
All-cause mortality	1191	2.63 (2.48–2.80)	436	7.33 (6.63–8.03)	518	1.60 (1.46–1.74)
Any major bleeding	1152	2.55 (2.40–2.70)	325	5.46 (4.86–6.07)	593	1.83 (1.68–1.98)
Intracranial	223	0.49 (0.43–0.56)	53	0.89 (0.65–1.14)	125	0.39 (0.32–0.46)
Gastrointestinal	393	0.87 (0.79–0.96)	119	2.02 (1.65–2.39)	206	0.64 (0.55–0.73)
Other	630	1.41 (1.30–1.53)	183	3.16 (2.69–3.63)	313	0.98 (0.87–1.09)
Any thromboembolism	1578	3.49 (3.31–3.66)	405	6.81 (6.13–7.49)	910	2.81 (2.62–2.99)
Arterial	899	2.05 (1.91–2.18)	220	3.82 (3.30–4.33)	536	1.70 (1.56–1.85)
Myocardial infarction	662	1.49 (1.37–1.61)	170	2.92 (2.47–3.37)	371	1.16 (1.04–1.29)
Venous	66	0.15 (0.11–0.18)	25	0.42 (0.25–0.59)	31	0.10 (0.06–0.13)

Results subdivided in patients with iTTR below 60% or over 70%. Results presented in numbers of patients and complication per treatment year, with 95% confidence interval (CI)

Excessive alcohol use almost tripled the risk of having an iTTR < 60% (odds ratio (OR) 2.72; CI 2.27–3.24, p < 0.001). Concomitant chronic obstructive pulmonary disease (COPD), renal failure or dementia rendered an almost 50% higher risk of poor iTTR outcome (OR 1.45; CI 1.33–1.59, OR 1.47; CI 1.29–1.68, and OR 1.47; CI 1.02–2.12, respectively). Patients with diagnosed anaemia had 36% higher risk of having an iTTR below 60% (OR 1.36; CI 1.22–1.51). AF patients with a history of fall or with comorbidities such as hypertension, diabetes or cancer had over 20% higher risk of poor iTTR outcome [ORs between 1.21 and 1.25 (Table 3)].

**Table 3:** Multivariable regression analysis showing baseline factors associated with an individual TTR below 60% among patients with atrial fibrillation instituted on warfarin treatment

	OR	95% CI for OR	p
Age	0.99	0.98–0.99	< 0.001
Female sex	0.98	0.93–1.04	0.481
Stroke	0.85	0.79–0.91	< 0.001
TIA	0.91	0.82–1.00	0.056
Hypertension	1.25	1.18–1.32	< 0.001
Diabetes mellitus	1.25	1.16–1.33	< 0.001
Myocardial infarction	1.07	1.00–1.14	0.045
Cancer	1.21	1.11–1.31	< 0.001
Chronic obstructive pulmonary disease	1.45	1.33–1.59	< 0.001
Renal failure	1.47	1.29–1.68	< 0.001
Excessive alcohol use	2.72	2.27–3.24	< 0.001
Dementia	1.47	1.02–2.12	0.038
Liver disease	1.27	0.97–1.67	0.083
History of fall	1.23	1.11–1.35	< 0.001
Anemia	1.36	1.22–1.51	< 0.001
Previous major bleeding	1.00	0.85–1.16	0.971
Previous gastrointestinal bleeding	0.99	0.82–1.20	0.905
Previous intracranial bleeding	1.21	0.95–1.55	0.116

*OR* odds ratio, *CI* confidence interval

Previous stroke and higher age were the only baseline variables with statistically significant association with higher warfarin treatment quality, iTTR > 70% [OR 1.11; CI 1.04–1.18 and OR 1.01; CI 1.01–1.02 (Table 4)].

**Table 4:** Multivariable regression analysis showing baseline factors associated with an individual TTR over 70% among patients with atrial fibrillation started on warfarin treatment

	OR	95% CI for OR	p
Age	1.01	1.01–1.02	< 0.001
Female sex	0.99	0.95–1.05	0.826
Stroke	1.11	1.04–1.18	0.001
TIA	1.09	1.00–1.19	0.062
Hypertension	0.85	0.81–0.89	< 0.001
Chronic heart failure	0.80	0.76–0.84	< 0.001
Diabetes mellitus	0.81	0.76–0.86	< 0.001
Myocardial infarction	0.94	0.89–1.00	0.049
Cancer	0.84	0.78–0.91	< 0.001
Chronic obstructive pulmonary disease	0.67	0.62–0.73	< 0.001
Renal failure	0.65	0.58–0.74	< 0.001
Excessive alcohol use	0.39	0.32–0.47	< 0.001
Liver disease	0.87	0.66–1.14	0.308
History of fall	0.81	0.74–0.89	< 0.001
Anemia	0.74	0.67–0.82	<0.001
Previous major bleeding	0.97	0.84–1.12	0.699
Previous gastrointestinal bleeding	0.99	0.83–1.18	0.924
Previous intracranial bleeding	0.86	0.69–1.08	0.194

*OR* odds ratio, *CI* confidence interval

## Discussion

Warfarin treatment quality is of utmost importance to obtain optimal efficacy and safety. When instituting warfarin treatment, poor treatment quality measured by TTR can be predicted using the SAMe-TT_2_R_2_ score [5], or for an elderly population by using the PROSPER score [6]. However, these scores are developed in a low-TTR setting, predictors for poor and optimal TTR is likely to vary in countries with different TTR levels. In Sweden, the TTR level is above 70% in clinical practice, which is high by international comparison [2, 3].

We here present a large, nationwide retrospective register study with 28,011 AF patients, newly instituted on warfarin. During the study period DOACs were not available in Sweden in clinical practice, which otherwise could have biased the results. With in total 45,249 treatment years on warfarin and a mean TTR of 68%, excessive alcohol use was shown to be the most significant factor for poor INR control in our cohort, almost tripling the risk. Excessive alcohol use is an obvious reason for poor warfarin compliance and is a risk factor for bleeding in the HAS-BLED score [13]. However, excessive alcohol use is not part of the SAMe-TT_2_R_2_ score or the PROSPER score. Patients with known excessive alcohol use are commonly nowadays switched from warfarin to DOACs, despite little knowledge on compliance to DOACs in this patient group.

Regrettably, we cannot confirm the higher risk of poor TTR conferred by tobacco use due to lack of such information in our dataset. However, we do confirm that medical history is an important risk factor when AF patients with diagnosed hypertension, diabetes mellitus or previous myocardial infarction all had an increased risk for poor iTTR. We identified two other moderate risk factors of poor outcome in history of fall and cancer. Diagnosed renal disease was associated with an increased risk for poor TTR of 47%, within the same range as when coexisting diagnosis of COPD or dementia. These three risk factors are also not mentioned in the SAMe-TT_2_R_2_ score, however renal dysfunction is one of the most influential predictors of TTR in the risk score PROSPER.

Not all concomitant disorders are associated with poor INR control though. History of stroke—a risk factor for poor INR control in SAMe-TT_2_R_2_—is in our cohort instead associated with a good INR control. One can imagine that AF patients who have experienced embolic stroke are more compliant to their oral anticoagulant treatment, than AF patients who are treated with a primary prophylactic intent.

Our results shows an increased risk for all-cause mortality and major bleeding in patients with iTTR < 60%. Identifying those patients in forehand is therefore important. In patients with excessive alcohol use, the single strongest predictor for poor iTTR, it is however not clear if they would fare better on DOACs instead. In a subanalysis of the ROCKET-AF, there was a tendency towards lower iTTR in patients with excessive alcohol consumption [14], but no information of how these patients fare on DOACs. Excessive alcohol consumption both constitutes a risk factor for AF [15] and is an independent risk factor for stroke in low risk patients with AF [16], further studies on best choice of oral anticoagulation treatment for this patient group are needed.

Since this is a retrospective register study, we cannot confirm causality and the results should therefore, despite the large study population, be interpreted carefully. Some important background medical criteria, like tobacco consumption and medication such as antiarrhythmic are not included in our study.

## Conclusion

In a well-managed warfarin therapy setting for patients with non-valvular AF, excessive alcohol use is the greatest predictor of poor INR control. Many other concomitant vascular and organic specific disorders (such as hypertension, chronic obstructive disorder and renal failure) as well as variables of frailty (dementia, history of fall and cancer) are more frequently associated with poor INR control. For these patients one could consider alternative treatment options like DOACs. Patients with previous stroke were more likely to have good INR control.

## Electronic supplementary material

Below is the link to the electronic supplementary material.

Supplementary material 1 (DOCX 66 KB)
