# Supplementary material for: Predictors for INR-control in a well-managed warfarin treatment setting
Source: J Thromb Thrombolysis. 2018 Nov 8;47(2):227–32. doi: 10.1007/s11239-018-1765-4 (PMC6394450; doi:10.1007/s11239-018-1765-4)
Supplement: Supplementary file 1 — Supplementary material 1 (DOCX 66 KB) [file 11239_2018_1765_MOESM1_ESM.docx]

Appendix

| **Baseline characteristics** | **ICD-10 code or surgical procedure code** |  |
| --- | --- | --- |
| Stroke | I63-I64, I693–I694 |  |
| TIA | G450–G453, G458, G459 |  |
| Hypertension | I10–I15 |  |
| Chronic heart failure | I50, I110, I130, I132 |  |
| Diabetes Mellitus | E10-E14 |  |
| Myocardial infarction | I21–I22, I252 |  |
| History of fall(s) | W00–W19 |  |
| Cancer | C10–C90 |  |
| Chronic obstructive pulmonary disease | J40–J70 |  |
| Anemia | D50, D510, D513, D518, D519, D52, D53, D55, D560, D561, D562, D568, D569, D570, D571, D572, D588, D589, D59, D60, D61, D62, D63, D64 |  |
| Any major bleeding | I60–I62, S064–S066, R04, K250, K252, K254, K256, K260, K262, K264, K266, K270, K272, K274, K276, K280, K282, K284, K286, K922, K290, I850, I983, K625, D629, R589 |  |
| Gastrointestinal bleeding | I850, I983, K250, K252, K254, K256, K260, K262, K264, K266, K270, K272, K274, K276, K280, K282, K284, K286, K625, K920–922 |  |
| Intracranial bleeding | I60-I62, S064–S066 |  |
| Renal failure | I120, I131-I132, N17-N19, DR016, DR024, KAS00, KAS10, KAS20 |  |
| Excessive alcohol use | F10, K70, T51, Y90, Y91, E244, G312, G621, G721, I426, K292, K860, O354, Z714 |  |
| Dementia | F00-F03 |  |
| Liver disease | K70–K77, JJC, JJB | |
| Vascular disease (V in CHA_2_DS_2_-VASc**)** | I21–I22, I252, I70-71 | |
|  |  |  |
|  |  |  |
| **Endpoint definitions** |  |  |
| **Bleedings** |  |  |
| Intracranial | I60-I62, S064–066 |  |
| Gastrointestinal | I850, I983, K250, K252, K254, K256, K260, K262, K264, K266, K270, K272, K274, K276, K280, K282, K284, K286, K625, K920–922 |  |
| Other | D500, D508-509, D629, H365, H922, N02, N938–939, R04, R310 |  |
| **Thromboembolisms** |  |  |
| Arterial (Stroke / TIA / peripheral embolism) | I63-64, I74, G450-453, G458, G459 |  |
|  |  |  |
| Myocardial infarction | I21–22, I252 |  |
| Venous | I26, I636, I676, I80–82 |  |
